# The Lung–Kidney Axis: A Coordinated Regulation of Oxygen Sensing and Erythropoiesis

**DOI:** 10.3390/biomedicines14040886

**Published:** 2026-04-13

**Authors:** Ahmed Mansour Al Rajeh

**Affiliations:** Department of Respiratory Therapy, College of Applied Medical Sciences, King Faisal University, Al-Ahsa 31982, Saudi Arabia; amalrajeh@kfu.edu.sa

**Keywords:** erythropoiesis, erythropoietin, renin angiotensin

## Abstract

The lung–kidney axis forms an important physiologically integrated system which controls multiple essential functions of the body. An important observation of this interaction is tissue oxygenation and erythropoiesis, a vital process that involves erythropoietin (EPO) release by the kidney to bring red cell production into the bone, while pulmonary gas exchange ensures adequate oxygen delivery to the cells. Subsequently, the lung–kidney activation of the renin angiotensin system (RAS) influences vascular tone, blood pressure, and tissue perfusion, influencing the delivery of oxygen and the body’s requirement for erythropoietin. Additionally, beyond oxygen sensing, studies have evidenced the role of hypoxia-inducible factors (HIFs), inflammatory mediators, endothelial signaling pathways and iron availability. These modulate erythropoietin production, which enhances the process of erythropoiesis and arterial oxygen balance. Localized variations in renal oxygen levels together with hemodynamic control mechanisms enable the body to produce erythropoietin independently from systemic hypoxia conditions. This concept emerged to include the renal oxygen extraction fraction (OFE) and intrarenal microvascular shunting with perfusion oxygen coupling in governing EPO production. The present review refines the traditional knowledge to further expand our understanding of the lung–kidney axis regulating the process of erythropoiesis and arterial oxygen content. The integrative framework demonstrates that pulmonary arterial oxygenation and renal oxygen sensing together with bone hematopoietic responses operate as a unified system which maintains both oxygen equilibrium and hematopoietic balance throughout the body.

## 1. An Overview of the Lung–Kidney Axis in Systemic Homeostasis

**Figure 1:**
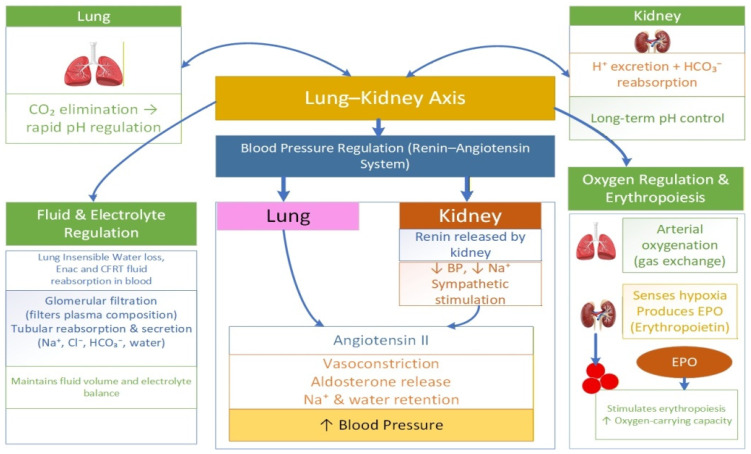
Crosstalk between lungs and kidneys in homeostasis.

The lung–kidney axis operates as a coordinated functional network regulating some of the vital functions of the body beyond acting as an autonomous organ system in maintaining homeostasis. The two systems interconnect through multiple regulatory domains, integrating pulmonary, renal and cardiovascular systems. This integration acts through various intricate neurohormonal and biochemical pathways. Central to this coordinated axis is the regulation of blood pressure and fluid balance control. Blood pressure regulation depends on the renin–angiotensin–aldosterone system (RAAS) and the counter regulatory measures of the ACE2/angiotensin-(1–7) system [1]. Together, these regulatory systems function to govern vascular tone and sodium balance to keep blood pressure stable. Equally critical is the role of the lung–kidney axis in maintaining acid–base balance. The lungs maintain systemic pH, acting immediately through their ability to eliminate carbon dioxide. Meanwhile, the kidneys maintain long-term pH stability through their role in tubular hydrogen excretion and bicarbonate reabsorption. This precise regulation maintains the pH in a steady state under variable metabolic and respiratory conditions [2]. In addition, another important integrating aspect of the lung–kidney axis is their involvement in fluid and electrolyte balance. The kidneys determine plasma composition through glomerular filtration and precise tubular reabsorption and secretion processes, thereby maintaining systemic electrolyte homeostasis [3]. Pulmonary hemodynamics and vascular permeability govern the distribution of intravascular and interstitial fluid [4]. The lungs enable insensible water loss through breathing. The epithelial sodium channel (ENaC) helps clear alveolar fluid through sodium transport followed by osmotic fluid drag from alveoli to the blood stream or interstitium. The lungs generate osmotic gradients for maintaining alveolar dryness, which enhances gas exchange through their controlled sodium and chloride transport system through ENaC and cystic fibrosis transmembrane conductance regulator (CFTR) channels. Furthermore, the lymphatic system removes excess fluid around alveoli and thus prevents pulmonary edema from developing [5,6]. Arterial oxygen and tissue oxygen signify additional key mechanisms in lung–kidney coordination [7]. The lungs provide arterial oxygenation through gas exchange, while the kidneys produce EPO in response to renal blood flow variation and hypoxic signals. EPO stimulates erythropoiesis and raises the blood’s oxygen-carrying capacity [8]. The synchronized activities of these processes demonstrate that the lung–kidney system operates as an integrated interdependent physiological network maintaining homoeostasis by regulating vital function; see Figure 1. This present study aims to further elaborate on and refine our understanding of the complex and dynamic interaction of this lung–kidney network. The predominant emphasis is on erythropoiesis, arterial oxygen balance and tissue oxygenation.

### Literature Selection Criteria

We conducted a comprehensive, web-based literature search that systematically examined major scientific databases, including PubMed, Scopus and Web of Science, to find relevant research studies about renal–respiratory crosstalk with regard to erythropoiesis and oxygen homeostasis. The search spanned databases covering studies published between 2004 and 2026. Keywords such as “erythropoiesis” and the Medical Subject Headings (MeSH) term “*lung kidney axis*”, combined with “*renin angiotensin aldosterone system*”, as well as MeSH terms like “*hypoxia-inducible factors*”, “nitric oxide”, “renal oxygen extraction”, and “blood viscosity” in conjunction with “renal involvement”, “*iron involvement*” *AND* “*endothelial signaling pathways*”, “*microvascular shunting*” *AND* “*bone marrow*”, and “*perfusion oxygen delivery coupling*”, were used to identify relevant studies.

## 2. The Lung–Kidney Axis in Oxygen Balance and Erythropoiesis

Erythropoiesis is a mechanism that causes red blood cell (RBC) production and affects the oxygen-carrying capacity of blood to sustain appropriate oxygen levels in the body tissues. The lungs, kidneys, and bone marrow function as a system operating through pulmonary gas exchange, renal oxygen sensing and hematopoietic regulation to maintain oxygen transportation and red blood cell balance. The lungs and kidneys work together to control RBC production through coordinated oxygen sensing and hormonal feedback mechanisms. This feedback ensures that red cell oxygen loading in the lungs, the oxygen-carrying capacity of blood and tissue oxygen perfusion remain balanced to prevent hypoxic conditions. The process of alveolar pulmonary gas exchange establishes systemic oxygen homeostasis through alveolar ventilation and diffusion, thus maintaining arterial oxygen saturation (SaO_2_) and the partial pressure of oxygen (PaO_2_) [9,10]. Any impairment in alveolar ventilation and diffusion leads to decreased oxygen delivery to the body, resulting in lower renal tissues oxygenation [11]. Meanwhile, kidneys function as the main oxygen detectors and endocrine systems for controlling red cell production. The kidneys constantly monitor systemic oxygen availability through changes in renal tissue oxygen tension (pO_2_), which reflects the arterial oxygen content (CaO_2_). Since the kidneys ensure high blood flow but maintain low oxygen extraction rates, any reduction in arterial oxygen levels causes substantial drops in renal cortical pO_2_ [12].

### 2.1. Oxygen Homeostasis: From EPO Production to Erythroid Signal Transduction

The kidneys maintain oxygen balance through their release of erythropoietin (EPO), a glycoprotein hormone secreted by special renal erythropoietin-producing cells. Erythropoietin adjusts red blood cell production to meet oxygen needs, preventing hypoxia [13]. EPO is a 30 kDa glycoprotein that facilitates normal erythropoiesis by enhancing the proliferation and differentiation of bone marrow erythroblasts. Erythropoietin facilitates its action through the EPO receptor (EPOR). EPOR is a 65 kDa glycoprotein that belongs to the class 1 cytokine receptor family expressed on red blood progenitor cells. EPO, through interactions with EPOR, plays a dominant role in the rapid expansion of red blood cell erythroid progenitors in response to hypoxic stimuli [14,15]. Recent studies have shown that EPOR activation by its ligand creates homodimers, initiating a chain of signal transduction that involves JAK2 and STAT5 phosphorylation [16]. The latter enters the nucleus and drives the transitional program for erythroid proliferation and differentiation [9]. STAT5 promotes the activation of transcription factors GATA and KLF; these regulate hemoglobin synthesis and erythroid maturation and hence influence erythropoiesis [9]. Erythropoietin production requires a two-step oxygen sensing system which connects oxygen delivery to the body with molecular transcriptional regulation. The first step requires arterial oxygen content (CaO_2_) to be converted into the kidney’s tubulointerstitial oxygen tension (pO_2_), a parameter determined by renal blood flow and hemoglobin levels and local metabolic oxygen consumption rates. The second step involves specialized renal EPO-producing (REP) interstitial fibroblast-like cells that detect local pO_2_ changes through the hypoxia-inducible factor (HIF) signaling pathway. The expression of EPO is significantly upregulated by HIF under hypoxic conditions [17].

### 2.2. Role of Hypoxia-Inducible Factor (HIF) in the Regulation of Erythropoiesis

**Figure 2:**
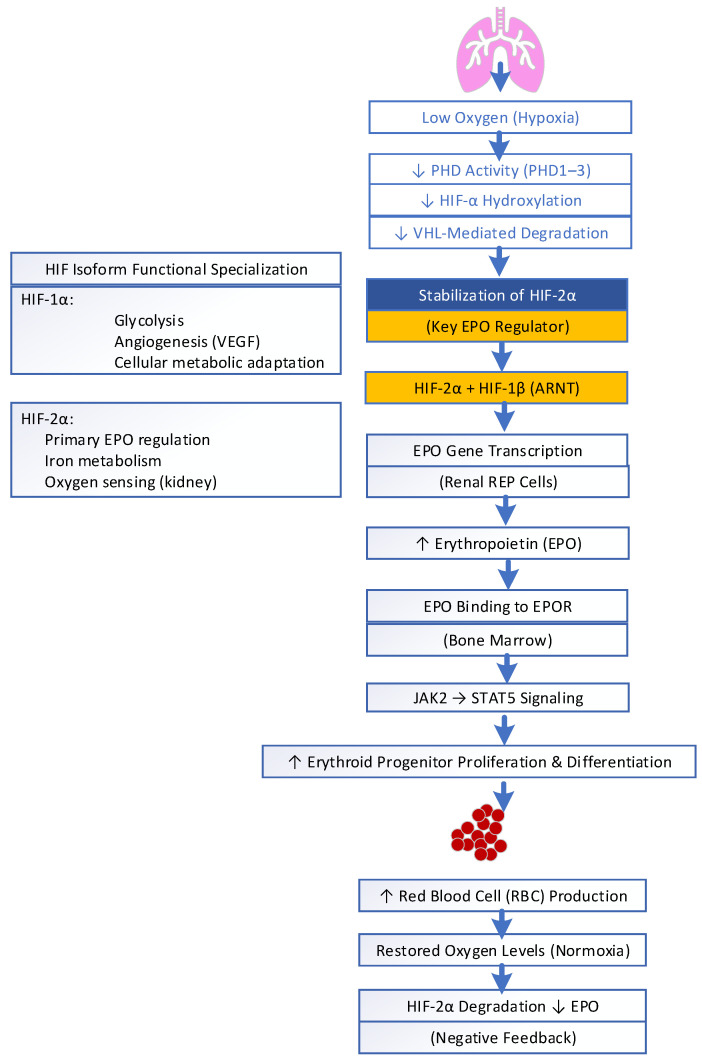
Hypoxia-inducible factor (HIF) in the regulation of erythropoiesis.

HIF is predominantly involved in the regulation of renal EPO. It is a master regulator for managing oxygen homeostasis. HIF, particularly the HIF-1α subunit, is produced and found in virtually all nucleated mammalian cells, primarily in vascular endothelial cells and specific parenchyma cells of organs like the heart, lungs, and kidneys. HIF is a central intracellular transcription regulator that enables cells to sense and adapt to variations in oxygen levels. HIF is a heterodimer consisting of an oxygen-sensitive α subunit continuously being synthesized in the cytoplasm and a constitutive β subunit, also known as aryl hydrocarbon receptor nuclear translocators (ARNTs). Three isoforms of HIF-α exist (HIF-1α, HIF-2α, or HIF-3α), among which HIF-2α is the primary transcriptional regulator of EPO expression and synthesis in renal REP cells; see Figure 2. Under normal conditions, HIF-α subunits are hydroxylated by oxygen-dependent prolyl hydroxylase domain enzymes (PHD1–3). This involves ubiquitination and proteasomal degradation via the von Hippel–Lindau (VHL) pathway. When renal tissue oxygenation falls, PHD activity is inhibited, allowing for the stabilization of HIF-2α. Recent studies have shown that stabilized HIF-2α translocates to the nucleus and forms a functional transcription factor with the HIF-1β subunit to regulate hypoxia-responsive genes, leading to EPO production [18]. This increase in EPO production stimulates erythroid progenitor proliferation and differentiation in the bone marrow, augmenting erythropoiesis and the oxygen-carrying capacity of blood. Once the hemoglobin levels rise and renal tissue oxygenation is restored, stabilized HIF-2α declines, leading to the downregulation of EPO production [19,20].

Recent studies have shown that, rather than controlling EPO gene expression, HIF-1α also manages cellular metabolic adaptation and angiogenesis (VEGF), glycolysis, and iron metabolism [21]. The HIF-2α–EPO–Hb axis activity varies with hypoxic duration and tissue type. Any abnormal function leads to anemia, polycythemia and tumors due to access activity [22]. Balance between these two isoforms is important for metabolic and vascular homeostasis. Dysregulation is linked to cancer, ischemia, and inflammatory diseases. Inhibiting HIF-2α activity with Belzutifan provides clinical utility in VHL-associated tumors [22]. Under pathological conditions, chronic HIF overactivation during sustained hypoxia or inflammation (e.g., chronic lung disease, CKD, cancer) promotes abnormal angiogenesis, fibrosis, vascular remodeling, erythrocytosis, pulmonary hypertension, and tumor progression, whereas insufficient HIF activation, especially in renal pathology, reduces EPO production, leading to anemia due to impaired hypoxic adaptation [21,23,24,25]. Hypoxia enhances renal HIF-2α-induced erythropoietin production and red cell formation, but persistent inflammation, oxidative stress and cytokines reduce EPO effectiveness and ineffective erythropoiesis. Acute lung injury occurs due to inflammation, increased alveolar permeability, and epithelial–endothelial damage. HIF mediates a dual effect in response to hypoxia in acute lung injury: it reduces inflammation and encourages vascular repair. Nevertheless, excessive activation increases vascular permeability, inflammatory cell deposition, and fibrosis. Consequently, HIF-1 is a promising yet complex novel therapeutic target in both acute kidney injury and acute lung injury that requires precise modulation [26,27].

REP cells are highly sensitive to hypoxia in renal interstitial fibroblasts; these cells lose their ability to synthesize EPO when they transform into myofibroblasts, leading to observed anemia in chronic kidney disease patients. This loss is linked to DNA methylation of the *Epo* and *Epas1* (HIF-2α) promoters, suppressing HIF-2α-driven EPO expression. This transformation may be reversible after injury resolution. Correspondingly, the loss of EPO responsiveness in chronic hypoxic disorders such as COPD due to systemic inflammation mediated erythropoietin resistance and HIF- dependent oxygen sensing pathways [28]. The recent identification of the HIF pathway and development of HIF-prolyl hydroxylase inhibitors (HIF-PHIs) represent major recent advances in the treatment of anemia [29,30]. Newer discoveries of HIF-PHIs used in CKD-related anemia increase hemoglobin by stimulating endogenous EPO production and enhancing iron availability. But concerns persist regarding potential risks, which include cardiovascular events, tumor progression, and hyperkalemia [30]. Studies have also shown that selective HIF-2α stabilizers (e.g., *roxadustat*, *daprodustat*, *vadadustat*) have been developed therapeutically to induce EPO production in patients with COPD- or CKD-associated anemia. Hypoxia-inducible factor prolyl hydroxylase inhibitors (HIF-PHIs) therapeutically exploit endogenous hypoxia signaling to stimulate EPO production while improving iron utilization, offering a unified approach to correcting anemia in chronic kidney and pulmonary diseases [30,31].

### 2.3. Lung–Kidney Axis on Erythropoietin Production: Linking Local Pulmonary Signaling to Systemic Erythropoiesis and Respiratory Function

Furthermore, recent research has revealed that renal erythropoietin (EPO) production is not autonomous, even though kidneys are the primary source of circulating EPO. The liver also serves as an important source of erythropoietin (EPO), contributing approximately 10% to adults and acting as the primary site during fetal development. Recent studies indicate that, under conditions such as hypoxia or renal dysfunction, hepatic EPO production can be upregulated via HIF2α activation in hepatocytes, thereby supporting erythropoiesis as a compensatory response [22].

Emerging research studies show that the lungs also express low but measurable levels of EPO. However, pulmonary EPO production is quantitatively minor and creates small amounts of EPO, which do not drive the main process of blood cell production. EPO receptors (EPO-Rs) are widely expressed in alveolar type II epithelial cells, bronchial epithelial cells, vascular endothelial cells, and pulmonary artery smooth muscle cells [32,33,34]. Pulmonary EPO signaling maintains local HIF pathways’ oxygen sensing and protection of alveolar epithelial tissue and may indirectly enhance renal EPO output during hypoxic stress [32,35]. Additionally, EPO exerts non-hematopoietic effects protecting the pulmonary endothelium and reducing oxidative stress, showing a bidirectional relationship where lung ventilation perfusion regulates renal EPO, and renal EPO helps maintain pulmonary vascular health [26,36]. The lungs also contribute to circulating growth factors and cytokines (e.g., VEGF, IL-6) that influence EPO release and erythroid maturation.

Erythropoietin (EPO), beyond its role in red blood cell production, also acts as a potent tissue-protective, anti-apoptotic, and anti-inflammatory agent through the EPOR/βcR heteroreceptor [37,38]. It activates the JAK2/STAT5 and PI3K/Akt pathways to promote cell survival and tissue repair while inhibiting NF-κB-mediated inflammation, thereby reducing pro-inflammatory cytokines such as TNF-α and IL-6. EPO also preserves endothelial barrier integrity, decreases vascular permeability, and limits oxidative stress by reducing reactive oxygen and nitrogen species, collectively protecting lung and endothelial tissues [32]. Studies on anesthetized animals showed that erythropoietin EPO acts also as a neuromodulator in the central nervous system, enhancing breathing through its effects on ventilatory drive, respiratory rate, tidal volume and minute ventilation. Studies recently also reported that neural EPO reduces early-life stress-induced respiratory disturbances observed in males, thereby reducing abnormal breathing patterns [39]. EPO also enhances the hypoxic ventilatory response through interactions with the brainstem respiratory centers and carotid bodies. Clinical studies in hemodialysis patients receiving recombinant human EPO (rHuEPO) showed improved pulmonary performance, including increased maximum voluntary ventilation, forced vital capacity (FVC), and peak expiratory flow rate (PEFR). EPO furthermore reduces airway resistance through its ability to induce bronchodilation through nitric oxide-mediated pathways to prevent cytokine-induced bronchoconstriction and airway resistance, thus resulting in lung protection from injury and enhanced respiratory performance [40,41,42].

### 2.4. Bone Marrow Response and Expanded Hematopoietic Paradigm

Regardless of its production in the kidneys or lungs, erythropoietin (EPO) exerts its effects, binding primarily to erythropoietin receptors (EPORs) and stimulating erythroid progenitor cell proliferation and differentiation in bone narrow. The binding of EPO to EPOR homodimers activates intracellular signaling pathways, with the JAK2–STAT5 pathway serving as the primary mediator of erythropoietin gene regulation [41,43]. The bone marrow therefore remains the central organ for EPO-induced erythropoiesis and systemic hematologic homeostasis. The spleen and liver also contribute as secondary (extramedullary) hematopoietic sites, particularly under stress conditions such as hypoxia, anemia, or bone marrow dysfunction, where they support additional erythropoiesis to maintain the oxygen-carrying capacity [44]. However, the recent evidence has broadened the traditional bone marrow-centered model of hematopoiesis. This evidence reveals that the lungs also exhibit an intrinsic hematopoietic capacity. Initial studies in animal models also demonstrated that the lungs serve as a reservoir for hematopoietic stem and progenitor cells (HSPCs) predominantly localized to the alveolar interstitium. These are involved in blood cell generation under normal physiological conditions and in response to stress or injury. Pulmonary HSPCs exhibit distinct gene expression profiles enriched for erythroid, megakaryocyte/platelet, and immune pathways. They demonstrate proliferative and multilineage abilities in experimental models [45]. Recent evidence shows that the adult human lung contains functional HSPCs, located mainly in the alveolar interstitium with a frequency comparable to bone marrow, that are capable of proliferation and engraftment. These lung-derived progenitors exhibit gene signatures enriched in immune, megakaryocyte/platelet, and erythroid pathways, indicating that the lung may serve as a specialized niche contributing to hematopoiesis. Hematopoiesis in the lungs is a new and emerging concept [45,46,47,48]. These findings expand the classical bone marrow-centric view of hematopoiesis to include the lungs as an active hematopoietic niche supporting both erythropoiesis and platelet production [9,10]. These discoveries broaden our understanding of oxygen homeostasis by situating the lungs both as a gas exchange organ and as having hematopoietic function, while redefining EPO as a multifunctional factor at the interface of pulmonary and renal physiology.

## 3. Acid–Base Regulation, Sympathetic Signaling, and Iron Metabolism in Erythropoiesis: A Link Within the Lung–Kidney Axis

### 3.1. Iron Metabolism in Erythropoiesis

**Figure 3:**
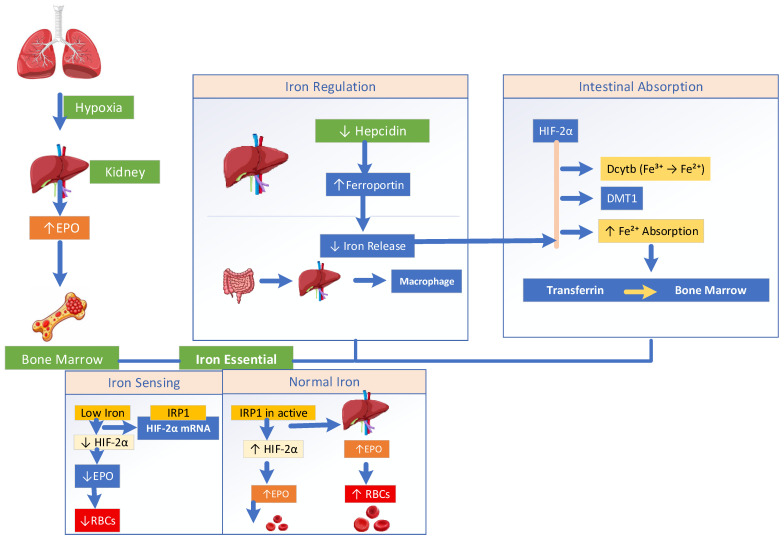
Iron metabolism in erythropoiesis.

Oxygen tension alone does not set the regulatory threshold for the process of erythropoiesis. The classical understanding of erythropoiesis has been redefined by advances establishing that renal oxygen sensing is linked to systemic iron metabolism; see Figure 3. Effective red cell production requires not only established adequate hypoxic-induced EPO signaling. The newer understanding demonstrates the coordinated availability of iron, predominantly governed by the hepcidin–ferroportin axis. Under hypoxic conditions, HIF2α enhances intestinal iron absorption by upregulating duodenal cytochrome b (Dcytb), which converts ferric iron (Fe^3+^) into ferrous iron (Fe^2+^). This ferrous iron is then transported into the cells via DMT1, thereby increasing iron availability for erythropoiesis [49]. Iron is consequently exported via ferroportin (FPN), enters the circulation bound to transferrin, whose expression is also HIF regulated, and is delivered to bone marrow to produce hemoglobin. Under low oxygen levels and low serum iron, the increased erythropoietic drive suppresses hepatic hepcidin production. Permitting greater ferroportin action causes greater iron release from enterocytes, hepatocytes, and macrophages to meet marrow need [50]. At the molecular level, iron availability directly modulates oxygen sensing and EPO gene expression. However, when intracellular iron is scarce, iron regulatory protein-1 (IRP1) binds to the 5′ untranslated region of HIF-2α mRNA, inhibiting its translation and attenuating hypoxia-induced erythropoiesis despite oxygen deprivation [51,52]. Iron shortage may also promote HIF-2α degradation even under hypoxic conditions [53]. Conversely, in iron-replete states, the formation of the iron–sulfur cluster on IRP1 prevents its interaction with HIF-2α mRNA, allowing efficient HIF-2α translation and appropriate EPO production.

#### 3.1.1. IRP Regulation of HIF-2α and Erythropoiesis

IRP1 and IRP2 regulate erythropoiesis and iron metabolism through the IRP/IRE system. IRP1 controls HIF2α translation in the kidneys, thereby modulating EPO production in response to oxygen availability. IRP2 ensures an adequate iron supply for hemoglobin synthesis in erythroblasts via TfR1. In conditions of IRP1 deficiency, increased HIF2α activity elevates endothelin-1 (ET-1) levels, leading to pulmonary vasoconstriction, pulmonary hypertension, cardiac hypertrophy, and fibrosis. Together, these mechanisms highlight the role of iron regulation in linking the lung–kidney axis with oxygen sensing and erythropoietic control [9,53,54,55,56,57]. Iron acts as a permissive regulator of erythropoiesis by controlling the stability of hypoxia-inducible factor 2α (HIF-2α), the key transcription factor for erythropoietin (EPO) [53]. When iron is sufficient, HIF-2α is degraded, limiting EPO production. Meanwhile, iron deficiency stabilizes HIF-2α, increasing EPO and promoting red blood cell formation, but only if iron is available for hemoglobin synthesis. In chronic inflammatory or hypoxemic conditions, this regulation is disrupted. Inflammation elevates hepcidin, a liver-derived hormone, which binds to ferroportin, the main iron exporter on enterocytes, macrophages, and hepatocytes, causing its internalization and degradation. This prevents iron release into the plasma despite adequate or increased body iron stores, creating a state of functional iron deficiency. Consequently, even with high EPO levels, erythropoiesis is impaired because iron is unavailable for hemoglobin assembly, leading to EPO resistance [52]. This maladaptive response contributes to anemia, commonly seen in chronic kidney disease and hypoxemic chronic lung disorders such as COPD. This is due to the combined effects of impaired pulmonary oxygenation, renal hypoxia signaling, inflammation, and disrupted iron homeostasis. This demonstrates that iron metabolism serves as a key link between pulmonary oxygen sensing and renal hormone control, emphasizing the lung–kidney axis as an iron-dependent system for preserving systemic oxygen balance [58].

#### 3.1.2. Expanding Paradigms: Erythropoiesis Under Iron Dysregulation

Recent studies have greatly expanded our understanding of erythropoiesis. Studies have revealed multiple critical factors and pathways that regulate red blood cell production under defective iron metabolism. This could be an important implication for treating anemia and other hematopoietic disorders. Metabolic regulation, such as the isocitrate pathway, has been shown to bypass hepcidin-induced aconitase inhibition during inflammatory anemia, restoring erythropoiesis. Novel growth factors and regulators have emerged, including erythroferrone (ERFE), which suppresses hepcidin to increase iron availability. GDF11 is a TGF-β family member that inhibits erythroid maturation, targeted to treat ineffective erythropoiesis. Polymeric human IgA1 stimulates erythropoiesis via transferrin receptor 1 to enhance EpoR signaling. At the transcriptional level, a minimal set of factors—Gata1, Tal1, Lmo2, and c-Myc (GTLM)—can directly convert cells into functional erythroid progenitors. Advances in in vitro production using induced pluripotent stem cells (iPSCs) have further enabled the large-scale generation of red blood cells. Detailed studies of terminal maturation and enucleation have collectively provided a comprehensive view of the complex, multi-layered regulation of erythropoiesis [59,60].

### 3.2. Acid–Base Balance as a Key Non-Hypoxic Regulator of Erythropoiesis

Recent studies highlight acid–base balance as another key non-hypoxic regulator of erythropoiesis. Physiologically, lungs rapidly remove CO_2_, while the kidneys provide a slower metabolic pH buffering system by synthesizing bicarbonate during acidosis and excreting hydrogen ions [61]. Experimental studies show that both metabolic acidosis and respiratory acidosis decrease the production of EPO. This occurs even with an insufficient intrarenal distribution of oxygen due to altered pH-induced inhibitory action on the renal EPO producing cells [62]. The HIF-2α pathways that respond to oxygen levels serve as the main control mechanisms for EPO production. The acidic environment inhibits the process by making the HIF pathway inefficient, leading to decreased EPO despite hypoxic conditions. Recent studies show that acidosis attenuates the hypoxic stabilization of HIF-1α by activating lysosomal degradation [63]. Chronic kidney disease (CKD) leads to metabolic acidosis that triggers kidney inflammation, fibrosis and neurohormonal systems. This occurs through maladaptive mechanisms which include the renin–angiotensin–aldosterone system and endothelin, impairing renal erythropoietin response to hypoxia. The combination of acidosis and chronic inflammation results in hepcidin elevation, which restricts iron access needed for hemoglobin production. The combination of impaired oxygen sensing and dysfunctional EPO signaling, together with inflammation and functional iron deficiency, leads to anemia development in CKD patients [64].

Clinically, the application of alkali therapy to treat acidosis leads to better metabolic conditions and improved kidney function, which creates favorable conditions for optimal erythropoiesis [65,66]. Research shows that acidic environments lead to decreased bone erythroid function and the development of EPO hyporesponsiveness [67]. This is coupled with inflammation and iron imbalance, which typically affect patients with CKD and chronic lung disease. Recent studies have shown that, in COPD, hypercapnia induces renal vasoconstriction and increases renovascular resistance, supporting the concept that CO_2_ retention can impose renal hemodynamic stress [68]. This CO_2_-mediated vasoconstriction is largely driven by a sharp increase in sympathetic tone, characterized by elevated circulating levels of norepinephrine. This results in a dose-dependent reduction in kidney blood flow and impairment of glomerular filtration [68]. Respiratory acidosis increases tubular bicarbonate reabsorption, raising renal oxygen consumption. This, in the context of pulmonary-driven hypoxemia, exacerbates medullary hypoxia. Acute rises in CO_2_ increase pulmonary vascular resistance, causing hemodynamic modifications responsible for reductions in the pressure gradient across glomeruli, raising the renal interstitial pressure. At the molecular level, congestion and acidosis can disrupt eNOS activity, leading to superoxide production and oxidative stress. This damages endothelium and promotes irreversible tubulointerstitial fibrosis [68]. COPD patients experience chronic CO_2_ retention, which leads to recurring acid–base imbalances. This can interfere with their kidneys’ natural functions, despite their condition of low oxygen levels and occasional high erythropoietin levels. The body experiences systemic pH changes when pulmonary gas exchange becomes impaired that could affect renal HIF-EPO signaling. This leads to damage to the renal microenvironment that stimulates bone marrow red cell production.

The extended viewpoint provides an explanation for why anemia develops in patients who suffer from chronic lung and kidney diseases despite hypoxic conditions. Lung function and systemic acid–base balance, together with their common effect on erythropoiesis, demonstrate how proper acid–base balance can enhance kidney EPO secretion beyond the traditional oxygen-dependent mechanism. This insight demonstrates that the acid–base balance serves as an essential non-hypoxic factor that controls erythropoiesis, together with the known effects of oxygen levels and iron supply [65,69].

### 3.3. Sympathetic Modulation of Erythropoietin Release and Erythropoiesis

Sympathetic neurohumoral signaling modulates erythropoietin (EPO) production and can influence renal EPO-producing (REP) cells [70]. The sympathetic nervous system controls EPO synthesis when carotid bodies and medullary chemoreceptors identify low oxygen levels. This leads to the activation of sympathetic outflow that controls renal blood flow and oxygen consumption, while also inducing EPO synthesis through β-adrenergic pathways. This neural mechanism establishes a connection between respiratory sensing and renal endocrine responses, thus adding another regulatory layer for erythropoiesis beyond the classical oxygen dependent mechanism [71]. Experimental studies have shown that epinephrine uses β2-adrenergic receptors to control HIF-2α signaling in the kidneys. This leads to increased Epo mRNA expression and greater EPO production [72]. The mechanism provides better support for erythropoietic adaptation under hypoxic conditions. The sympathetic regulation system also controls renal blood flow and renin–angiotensin pathway activity, leading to changes in oxygen detection and erythropoietin production [73]. Clinical evidence supports altered renal sympathetic nerve activity to corresponding erythropoietin (EPO) levels, indicating the influence of sympathetic tone on the process of erythropoiesis [70]. Recent studies have shown that epinephrine enhances hypoxia-induced EPO production through the activation of the β2-adrenergic receptor interacting with HIF-2α. In COPD, sympathetic activation functions as a compensatory mechanism by increasing EPO production and erythropoiesis in the hypoxic state [28]. Recent findings propose a model in which sympathetic stimulation serves as a positive neuroendocrine signal, linking pulmonary hypoxia to renal EPO production and subsequent bone marrow erythropoiesis [72], thereby strengthening the concept of a lung–kidney–bone marrow axis that preserves systemic oxygen homeostasis.

#### Sympathetic Overdrive and Loss of EPO Function

**Figure 4:**
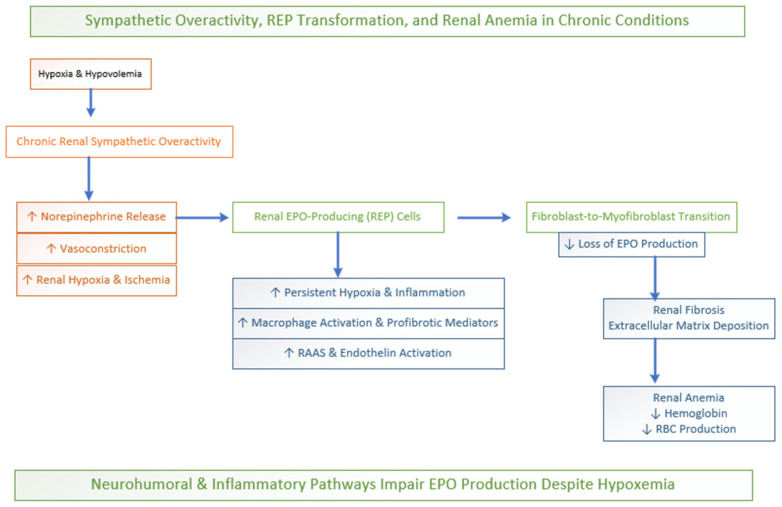
Sympathetic regulation of EPO.

In conditions of hypoxic stress and hypovolemia, the acute sympathetic nervous system prompts adrenal epinephrine release. This could enhance hypoxia-induced erythropoietin (EPO) production via the β-adrenergic receptor with the HIF-2α pathway in the kidneys; see Figure 4. This adaptive response accelerates red blood cell production and improves oxygen delivery. Thus, short-term sympathetic activation supports erythropoiesis and cardiovascular output. However, chronic or persistent activation may exert inhibitory effects on red cell production [72,74].

In chronic conditions such as chronic kidney disease and longstanding hypertension, persistent renal sympathetic overactivity leads to sustained vasoconstriction, microvascular dysfunction, and interstitial remodeling, thus ultimately impairing EPO production and contributing to renal anemia [75]. Chronic longstanding renal sympathetic nerve activity leads to norepinephrine release, which binds mainly to α-adrenergic receptors located on afferent and efferent arterioles, resulting in a substantial vasoconstriction-induced decrease in renal blood flow to the cortical region. The combination of this chronic hypoperfusion and the kidneys’ excessive oxygen creates a situation of prolonged oxygen deficiency inside the kidney. This hostile microenvironment poses a significant risk to renal erythropoietin-producing (REP) cells. REP cells are fibroblast-like cells located within the peritubular interstitium [68].

Prolonged ischemia, persistent inflammatory signaling, and sympathetic-mediated profibrotic pathways drive REP phenotypic transformation into matrix-producing myofibroblasts. As these cells adopt a fibrogenic role characterized by excessive extracellular matrix deposition, they progressively lose their capacity to synthesize erythropoietin [76]. Sympathetic overactivity further amplifies this process by modulating immune responses, including macrophage polarization and the release of profibrotic mediators and extracellular vesicles. This reinforces the fibroblast-to-myofibroblast transition and contributes to progressive renal fibrosis and anemia [77]. The functional decline of REP cells leads to the complete loss of the kidney’s ability to detect hypoxia, leading to the onset of chronic renal anemia; see Figure 4.

Recent studies show that REP cells in chronic kidney disease transform into myofibroblast-like cells which drive both renal fibrosis and progressive EPO production loss, leading to renal anemia. Correspondingly, sympathetic signaling often initiated by hypoxia in COPD can alter the phenotype of renal REP cells, shifting REP cells from erythropoietin secretion toward myofibroblast transformation [78]. In COPD, chronic CO_2_ retention and recurring acid–base disturbances further disrupt renal compensatory mechanisms, suppressing EPO production even when oxygen levels are low. The process is amplified by inflammatory cytokines (such as IL-6 and TNF-α) and iron dysregulation, which impair erythropoiesis and reduce EPO responsiveness. Persistent activation of the RAAS and endothelin systems further compromises renal endocrine activity. Together, the lung–kidney, neural and inflammatory “bridge” explains why anemia may persist in chronic lung disease despite hypoxemia. Consequently, neurohumoral and inflammatory mechanisms, and not oxygen deficiency alone, drive impaired EPO production [79,80].

### 3.4. Endothelium-Driven Vascular Signaling as a Non-Hypoxic Modulator of the Lung–Kidney Erythropoietic Axis

Nitric oxide (NO) is a key regulator of oxygen homeostasis. NO serves as an important signaling mediator linking pulmonary oxygen sensing to EPO production and subsequent bone marrow erythropoiesis. As an endothelial-derived factor, NO regulates pulmonary vascular tone to optimize ventilation–perfusion matching and arterial oxygenation [81]. Endothelial NO maintains low pulmonary vascular resistance and improves ventilation–perfusion matching by dilating vessels in well-ventilated areas, thereby enhancing oxygenation and reducing shunting, particularly in conditions such as ARDS [82,83]. While in the kidneys, NO modulates renal blood flow and tissue oxygenation, reduces vascular resistance, and promotes sodium excretion. NO also facilitates hypoxia-induced EPO production through cGMP signaling [84]. Under hypoxia conditions, NO modulates vascular tone in both organs, buffering excessive vasoconstriction and supporting tissue oxygen delivery. NO also provides cytoprotective effects by optimizing oxygen supply–demand balance [85,86,87]. Through these coordinated vascular actions, NO plays a key role in precisely regulating systemic oxygen delivery and red blood cell production [88]. Beyond intrinsic renal hypoxia sensing, endothelial-derived relaxing factors work in concert with renal oxygen sensing pathways to modulate oxygen delivery, HIF signaling, and erythropoietin synthesis [83,89,90]. This interplay highlights the critical role of endothelial signaling in linking pulmonary oxygen dynamics to renal EPO production and subsequent bone marrow erythropoiesis, emphasizing the integrated function in maintaining systemic oxygen homeostasis [91,92,93,94].

#### 3.4.1. Endothelial Nitric Oxide Signaling in Renal Oxygen Sensing and Erythropoietic Regulation

At the molecular level, shear stress and hypoxia activate endothelial nitric oxide synthase (eNOS), resulting in an increased production of nitric oxide (NO) and prostacyclin [95]. These signaling molecules elevate cGMP and cAMP levels in vascular smooth muscle. This leads to vasodilation that enhances ventilation–perfusion matching and increases arterial oxygen delivery. Experimental studies have further demonstrated that NO amplifies hypoxia-induced EPO mRNA expression and erythropoietin synthesis in the kidney via NO/cGMP-dependent pathways. However, the inhibition of nitric oxide synthase reduces EPO production. Together, these findings indicate that NO directly contributes to renal oxygen sensing mechanisms and modulates the HIF-mediated regulation of erythropoiesis [89,96]. Systemic oxygen levels are sensed by the kidney, where endothelial-derived nitric oxide (NO) and prostacyclin (PGI_2_) help maintain medullary blood flow and suppress hypoxic signaling. In addition, endothelin-1 (ET-1) and angiotensin II act alongside NO as counter-regulatory endothelial mediators, collectively modulating vascular tone and tissue oxygenation [84,97]. Hypoxia and inflammatory signals activate HIF-1α. HIF-1α stimulates ET-1 production, which promotes localized vasoconstriction through ETA receptors to redirect blood flow toward well-ventilated alveoli. Meanwhile, ET-1 and angiotensin II act on AT1 receptors to reduce peritubular capillary perfusion, increase mitochondrial oxygen consumption, and exacerbate intracellular hypoxia. This highlights the complex interplay of endothelial factors in regulating renal oxygen sensing and erythropoietin production [98,99,100,101].

**Figure 5:**
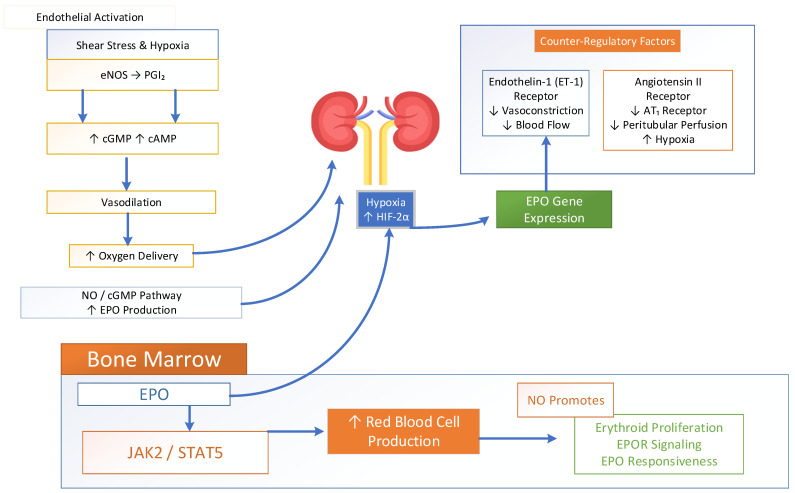
Endothelial nitric oxide-induced oxygen sensing and erythropoiesis.

The HIF-driven EPO secretion system activates JAK2-STAT5 signaling within bone marrow erythroid progenitors, which results in increased red cell mass; see Figure 5. This process creates a dynamic feedback system which uses endothelial vasoactive substances to convert pulmonary oxygen levels into renal gene expression and regulate systemic oxygen concentration. In the kidney, NO contributes to microvascular regulation and tissue oxygen distribution, indirectly affecting HIF-2α stabilization and erythropoietin gene activation. Recent studies have demonstrated that endothelial–stromal crosstalk regulates EPO production through nitrogen oxide-dependent changes in gene expression. This regulation is further modulated by microRNA signaling, particularly miR-210, along with coordinated metabolic adaptation [84,102,103,104]. Beyond renal effects, EPO itself stimulates endothelial NO production, creating a bidirectional regulatory loop that improves perfusion and oxygen delivery [105]. Studies further demonstrate that nitric oxide signaling is required for optimal EPO responsiveness in hematopoietic tissues. The inhibition of neuronal nitric oxide synthase reduces erythroid progenitor proliferation, EPOR signaling, and erythropoietic expansion, thus highlighting the direct role of NO in bone marrow adaptation to erythropoietic stress [89,106,107]. NO-dependent metabolic signaling promotes the expansion of early erythroid progenitors and helps maintain the balance between proliferation and differentiation.

The pulmonary and renal endothelial nitric oxide system functions as a vasodilator which enhances microvascular blood flow and improves oxygen delivery to tissues. It controls oxygen levels in renal cortex regions and stops the excessive activation of HIF-2α and abnormal EPO production during normal oxygen conditions [108,109]. Endothelin-1 (ET-1), in contrast, is the major endothelial counter-regulator. Endothelin-1 (ET-1) reduces blood flow through renal micro-vessels while increasing oxidative stress and localized hypoxia, leading to temporary HIF-2α stabilization and a subsequent EPO transcription increase. The balance between NO and ET-1 therefore acts as a vascular “rheostat”, finely controlling erythropoietic activity. In addition to this, endothelial sodium channels (ENaCs) and shear stress-sensitive signaling pathways function to regulate erythropoiesis through their impact on endothelial stiffness and nitric oxide availability and renal oxygen diffusion patterns [110]. The combined vasoactive signals directly control kidney oxygen detection and erythropoietin (EPO) synthesis through cGMP pathways, and oxidative stress operates for tissue oxygenation and erythropoiesis regulation [8,111].

#### 3.4.2. Endothelial NO–ET-1 Imbalance in COPD: Disruption of Lung–Kidney Erythropoietic Signaling

Under pathological states like COPD and kidney disease, the related hypoxia and inflammation lead to impaired endothelial signaling. The impaired signaling results in nitric oxide deficiency and an excess production of endothelin-1 and overactivation of the renin–angiotensin–aldosterone system [112,113,114,115]. The pathological profile increases kidney oxygen deficiency and creates HIF signaling disruptions, leading to excessive or insufficient EPO production and anemia [116]. Under chronic hypoxemic conditions such as COPD, disrupted endothelial NO signaling and hypoxia impair the coordinated lung–kidney axis. This results in maladaptive erythropoietic responses and bone erythropoiesis [68], emphasizing the role of nitric oxide as a dynamic signaling bridge that connects pulmonary oxygen sensing, renal endocrine adaptation, and bone marrow erythroid activity [117], thereby coordinating systemic oxygen homeostasis across the lung–kidney–bone marrow axis. Furthermore, NO counterbalances the vasoconstrictive actions of the renin–angiotensin system, helping to maintain renal hemodynamics [118]. The regulatory network which controls erythropoiesis becomes disrupted in COPD due to impaired pulmonary NO bioavailability, endothelial dysfunction and oxidative stress [119]. Thus, NO functions as a dynamic molecular link between pulmonary oxygenation, renal EPO production, vascular control mechanisms and bone marrow erythropoiesis [83]. These recent findings identify endothelial vasoactive signaling as a central integrative mechanism through which the lungs convey oxygen status to the kidneys [120], thus transforming vascular tone and tissue perfusion dynamics into the molecular control of EPO production and correspondingly coordinating bone marrow erythropoiesis to maintain systemic oxygen homeostasis.

## 4. Renal Oxygen Extraction Fraction (OEF) and Microvascular Shunting

The kidneys receive approximately 20–25% of cardiac output. Despite this high perfusion, renal tissue exhibits a low OFE that is 3% of the delivered oxygen, reflected by a small arterial–venous oxygen difference [121]. Therefore, renal tissue oxygenation is largely determined by how effectively the renal microvasculature delivers oxygen to meet the metabolic demands rather than arterial oxygen content alone [122,123]. Recent imaging studies and MRI investigations in acute kidney disease suggest that OEF and the renal metabolic rate of oxygen serve as an important functional parameter for evaluating renal function and renal oxygen content [124,125]. An important influence on renal oxygenation is Microvascular Oxygen Shunting, in which oxygen diffuses directly from arterial vessels to nearby veins before reaching capillaries and tubular cells [68]. This is prominently observed in renal medulla, where the countercurrent exchange system facilitates oxygen shunting, thus providing low oxygen tension in the medulla and increasing its vulnerability to hypoxia. Under pathological conditions such as COPD, pulmonary oxygenation decreases, leading to a drop in the arterial oxygen content that results in reduced renal tissue oxygen saturation, especially in the medulla [68,126]. In response, the kidney increases its OEF to maintain the tubular oxygen demand. However, microvascular oxygen shunting may limit tissue oxygenation, deteriorating medullary hypoxia. Under conditions of reduced local oxygen tension, renal interstitial cells normally activate HIF signaling pathways that stimulate the production of erythropoietin and accelerate erythropoiesis. However, prolonged renal hypoxia and structural injury to erythropoietin-producing cells, commonly observed in chronic kidney disease, can impair erythropoietin secretion, leading to reduced erythropoiesis and the development of anemia [19,127,128,129,130]. Thus, it is emphasized that, under chronic hypoxic conditions as in COPD, impaired pulmonary oxygenation reduces arterial oxygen content. This results in a decline in renal tissue oxygenation, predominantly in the medulla, which is highly susceptible to hypoxia. In this response, the kidney increases its OEF to meet tubular metabolic oxygen demands. This compensatory mechanism helps sustain essential renal functions despite the reduced oxygen availability. However, persistent and chronic hypoxic conditions can disrupt the adaptive response of normal renal functioning. Consequently, lung dysfunction becomes closely linked to altered renal hemodynamic and hypoxia. This interaction results in abnormal erythropoietic regulation.

## 5. Renal Hemodynamic Regulation (Perfusion–Oxygen Delivery Coupling)

The process of renal hemodynamic regulation establishes an essential connection between pulmonary function and the process of erythropoiesis. The two primary factors that determine blood circulation throughout the body are pulmonary vascular resistance and right ventricular function. Right ventricular dysfunction leads to increased central venous pressure. Under chronic pulmonary diseases, pulmonary hypertension and sustained hypoxic vasoconstriction increase pulmonary vascular resistance (PVR). The elevated resistance forces the right ventricle to generate a higher pressure in order to maintain adequate blood flow [126,131].

While kidneys normally receive approximately 20–25% of the cardiac output, oxygen extraction remains relatively low. Every slight decrease in the right ventricular output will result in decreased renal blood flow and renal tissue oxygenation. This decreases the arteriovenous perfusion gradient, resulting in impaired renal oxygenation despite normal arterial oxygen levels [132,133]. The renal autoregulatory mechanisms and tubule–glomerular feedback system usually maintain blood flow during changes in systemic blood pressure. The persistent reduced cardiac output causes impaired oxygen delivery to cortical and medullary areas [134,135,136]. The kidneys require adequate arterial oxygen content and sufficient blood flow to maintain proper oxygen levels. Even when arterial oxygen is normal, reduced renal perfusion limits oxygen delivery to both the cortex and medulla. The kidneys have a high metabolic demand, particularly for tubular sodium reabsorption. This results in a mismatch between oxygen supply and consumption, leading to relative intrarenal hypoxia. The resulting decrease in cortical oxygen tension is sensed by erythropoietin (EPO)-producing cells, which activate hypoxia-inducible factor (HIF)-mediated pathways and increase EPO secretion, thereby stimulating erythropoiesis in the bone marrow [126]. These decreases in renal oxygenation trigger hypoxia-induced erythropoietin synthesis [121].

The EPO-producing cells show a high sensitivity to oxygen levels. Minor interruptions in microvascular flow lead to HIF-2α activation and EPO production variations. The mechanisms demonstrate how pulmonary vascular abnormalities disrupt renal oxygen flow. Kidneys act as precise monitors that transform cardiopulmonary signals into hormonal control of erythropoiesis to maintain oxygen levels [68]. Pulmonary hemodynamic abnormalities can affect red blood cell production even in the absence of systemic hypoxemia [137]. Clinically, this helps explain why patients with cardiopulmonary disease may develop erythrocytosis from sustained renal hypoxic signaling. Conversely, anemia with chronic congestion, inflammation, or microvascular injury impairs EPO responsiveness despite normal arterial PaO_2_ [22,138]. Thus, the coupling of renal perfusion and oxygen delivery constitutes a critical hemodynamic component of the integrated lung–kidney–bone marrow axis, governing systemic oxygen homeostasis.

## 6. Renin–Angiotensin System as a Central Integrator of Erythropoiesis Regulation

The renin–angiotensin–aldosterone system (RAAS) is a key biochemical pathway linking pulmonary function, renal oxygen sensing, and bone marrow erythropoiesis. The classical pathway involves the release of renin from renal juxtaglomerular cells, which is cleaved by liver-derived angiotensinogen into angiotensin I. It is then converted into angiotensin II (Ang II) by the angiotensin-converting enzyme (ACE), abundantly expressed on pulmonary endothelial surfaces. This strategic localization places the lung in a central regulatory role, coupling systemic oxygen status to renal endocrine signaling and hence red cell production in the bone. Angiotensin II is a primary active component of the renin–angiotensin–aldosterone system. Emerging studies suggest that angiotensin II, beyond its traditional role in blood pressure regulation, induces renal vasoconstriction and generates relative intrarenal hypoxia, thereby enhancing HIF-dependent EPO production [1].

### 6.1. Angiotensin II Signaling in Erythropoietic Regulation: Translational Evidence

Experimental studies using a deglycosylation-coupled Western blot method demonstrated that Ang II enhances plasma EPO synthesis. This occurs through the upregulation of EPO expression and HIF-2α activity, followed by PHD2 manifestation in nephron segments of the renal cortex and outer medulla. Localization analyses further revealed that Ang II stimulates EPO expression in both proximal tubules and collecting ducts, particularly within intercalated cells, emphasizing the functional interplay between renin–angiotensin signaling and renal hypoxia pathways [139,140,141]. In addition to modulating renal EPO synthesis, Ang II functions as an erythropoietin secretagogue and a growth-promoting factor for erythroid progenitors. The activation of the AT1 receptor promoted the development of early erythroid progenitors. This occurs by stimulating EPO release and working synergistically with EPO signaling in the bone marrow. Ang II enhances erythroid proliferation and differentiation, eventually boosting red blood cell mass and supporting effective erythropoiesis [142]. Numerous studies have established the renin–angiotensin system as a key regulator of erythropoiesis [143]. RAS inhibition can lower hematocrit levels, leading to anemia, and compromise renal function, since angiotensin II is essential for controlling GFR and stimulating erythropoiesis [142]. Pharmacologic studies show that angiotensin-converting enzyme (ACE) inhibitors reduce plasma EPO concentrations. Patients receiving RAS blockers often develop anemia associated with lower circulating EPO levels [144]. Cole et al. reported that reduced plasma angiotensin II may be a key contributor to anemia in ACE-knockout mice [145,146]. Furthermore, angiotensin II has been demonstrated to elevate EPO mRNA levels in the renal cortex and increase HIF-2α mRNA expression in both the cortex and proximal tubules [140]. Studies also suggested that Ang II indirectly acts as a growth factor for myeloid erythroid progenitors, while Kim et al. reported that Ang II stimulates EPO production through AT1R-dependent Egr-1 activation via the p21Ras–MAPK/ERK signaling pathway in human renal 786-O cells.

Aldosterone was found to increase EPO and HIF-2α mRNA expression in tubule suspensions and was also reported in micro-dissected medullary thick ascending limbs and outer medullary collecting ducts. Further studies using advanced methods have explored the effects of aldosterone and its analog fludrocortisone on renal EPO production. Similarly, fludrocortisone enhanced EPO mRNA and protein expression in distal nephron segments, particularly in intercalated cells of the collecting ducts. This demonstrates that mineralocorticoid signaling directly modulates renal erythropoietin synthesis [147]. Aldosterone directly stimulates EPO production within the nephron. The prolonged suppression of aldosterone, such as with RAS-blocker therapy, may contribute to anemia. Since aldosterone drives tubular EPO synthesis, an impaired tubular function in chronic kidney disease can also lead to renal anemia. Activation of the renin–angiotensin system has been linked to secondary erythrocytosis in chronically hypoxemic patients with COPD, supporting the concept of an integrated lung–kidney–bone marrow network. This demonstrates that renal renin release, pulmonary Ang II generation, and bone marrow erythropoietic activity collectively regulate oxygen homeostasis under physiological conditions [141].

### 6.2. Renin–Angiotensin System Activity and Bone Marrow Erythropoiesis

Further investigations with RAS inhibitors demonstrate that Ang II signaling regulates the proliferation and differentiation of specific hematopoietic populations, particularly erythroid cells. These effects are mediated in part through the widely expressed Ang II type 1 receptor (AT1R) on erythroid cells. Ang II modulates both renal and bone marrow responses and can act directly on erythroid progenitor cells in the bone marrow [142]. Although initially recognized as a circulating hormone, angiotensin II is now understood to be produced locally in multiple tissues. The bone marrow contains all the components required for local Ang II synthesis and expresses both AT1 and AT2 receptors. Ang II has been detected in bone marrow cultures, and circulating angiotensinogen and renin may further enhance its local generation in vivo. Hematopoietic stem cells (HSCs, CD34^+^) express Ang II receptors, and the administration of Ang II in mice increases HSC numbers and stimulates myelopoiesis [143,146,148]. Additionally, Ang II promotes erythroid colony formation from both mouse bone marrow and human CD34^+^ cells, modulating physiological and pathological erythropoiesis primarily via AT1 receptor signaling [149]. The study by Kato et al. demonstrated that transgenic mice who express both renin and angiotensinogen develop erythrocytosis, while the mice that lack these genes show symptoms of anemia. Angiotensinogen-knockout mice experienced anemia treatment through ATII infusion. This demonstrated that the angiotensinogen–ATII pathway functions as the main mechanism for EPO production induced by ATII. These results indicate that EPO regulation is governed not only by hypoxia or anemia but also by renin–angiotensin system (RAS) signaling [145].

## 7. Dual Regulation of Renal Erythropoietin Production: Nephron-Derived RAS Signaling Versus Hypoxia-Driven Interstitial Activation

Novel evidence suggests that the kidneys possess dual EPO production systems: RAS-regulated production by nephron segments under normal conditions and hypoxia-induced production by interstitial fibroblast-like cells [147]. EPO has been reported to originate from FOXD1-expressing stromal cells, particularly in response to hypoxia or anemia. These include interstitial fibroblast-like cells, renin-producing cells, vascular smooth muscle cells, and mesangial cells. Anemia and low oxygen levels trigger EPO synthesis via the activation of HIF and HIF prolyl hydroxylase (PHD) pathways. HIF-2α expression is localized to interstitial cells rather than nephrons, supporting the role of fibroblast-like interstitial cells as the primary renal EPO-producing population [150,151]. Recent studies, however, suggest that HIF-2α is expressed throughout most nephron segments. EPO mRNA has been detected within these segments in several reports. Using in situ hybridization, under both baseline and hypoxic conditions, EPO is produced by cortical nephron segments, whereas interstitial cell-derived EPO is produced under hypoxic conditions [25]. This indicates that severe anemia or marked hypoxia is necessary to trigger EPO production from interstitial fibroblast-like cells. Collectively, under normal, non-stressed conditions, nephron segments are the primary source of EPO, regulated mainly by the renin–angiotensin–aldosterone system.

## 8. Conclusions

**Figure 6:**
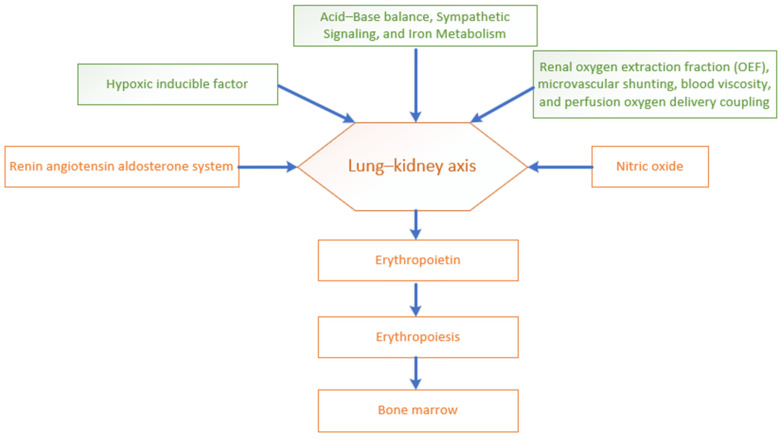
Multifactorial regulation of erythropoiesis by the lung–kidney axis: integration of HIF, RAAS, nitric oxide, and oxygen dynamics.

Regulating the process of erythropoiesis and oxygen balance represents a finely balanced system coordinated through the lung–kidney–bone marrow axis. This lung–kidney-induced erythropoiesis and oxygen balance integrates oxygen sensing, neuroendocrine control, iron metabolism, and vascular signaling to maintain tissue oxygenation. Pulmonary gas exchange regulates systemic oxygen content, while the kidneys detect oxygen saturation, leading to erythropoietin (EPO) production mainly through the hypoxic stimulation of HIF-2α signaling to maintain adequate oxygen delivery via the bone marrow. Erythropoietin release and erythropoiesis are not only influenced by hypoxia but also by iron metabolism, acid–base balance, sympathetic signaling, nitric oxide, endothelin-1, and the renin–angiotensin–aldosterone system (RAAS); see Figure 6. Additionally, the renal oxygen extraction fraction, renal microvascular shunting and perfusion–oxygen delivery modify renal hemodynamic and EPO production under varying oxygen statuses.

## Data Availability

No new data were created or analyzed in this study. Data sharing is not applicable to this article.
